# A New Method of Treating Syphilitic Bubo

**Published:** 1853-09

**Authors:** B. W. Hazlett

**Affiliations:** Wheeling, Virginia


*A New Method of Treating Syphilitic Bubo.* By B. W. HAZLETT, M. D., of Wheeling, Virginia.

To the Editors of the Medical Examiner:

GENTLEMEN,—Through the medium of your periodical allow me to notice a method of treating Suppurating Syphilitic Bubo, which I find not made mention of elsewhere. The rationale of treatment is familiar to surgeons in chronic abscess, &c., &c., but no use has been made of it in suppurating lymphatic of specific character. My plan is simply to inject with the common ear-glass syringe of the following formula:

℞ Argent nitrat. cryst. . . . . . gr. xxv.
Aquæ distillat. . . . . . . ℥i.
Mix.

Some little tact is necessary to bring the fluid in contact with the entire suppurating surface. This will be accomplished by care in the position of the patient, permitting the injection to gravitate to the extent of the bubo. If this is not effected easily, pressure from behind will drive the cautery to all the sinuses and interstices that exist. I am partial in opening (with the common thumb lancet) the bubo as soon as pus is separated, and immediately, on pressing out the virus, to administer the injection. In this manner I have seldom used more than *one* injection, and in cases where extensive sinuses exist, *two* are all-sufficient.

The advantage over the old method of destroying the skin with the potassa fusa, or excising with the knife, and waiting the tedious process of granulation, must be apparent, besides obviating the unpleasantness of confinement to bed.

